# Network Analysis of Eating Disorders Symptoms Co-occurring With Impulsive Personality Traits and Negative Mood States in Patients With Bulimia Nervosa

**DOI:** 10.3389/fpsyt.2022.899757

**Published:** 2022-05-18

**Authors:** Yan Chen, Lei Guo, Mengting Wu, Lei Zhang, Qianqian He, Yuchen Zheng, Lin Wu, Hui Zheng, Jue Chen

**Affiliations:** ^1^Shanghai Mental Health Center, Shanghai Jiao Tong University School of Medicine, Shanghai, China; ^2^Military Medical Psychology School, Air Force Medical University, Xi’an, China; ^3^Shanghai Key Laboratory of Psychotic Disorders, Shanghai Mental Health Center, Shanghai Jiao Tong University School of Medicine, Shanghai, China

**Keywords:** bulimia nervosa, anxiety, depression, impulsivity, network analysis

## Abstract

**Background:**

Bulimia nervosa (BN) is characterized by recurrent episodes of eating large amounts of food without control. Studies have found positive correlations of BN symptoms with impulsive traits and negative affect. However, the network relationship supporting BN symptoms is unclear.

**Methods:**

The study participants included female BN patients (146) and healthy controls (HCs, 146). The participants were matched for age. All participants completed the Eating Disorder Examination Questionnaire 6.0, Barratt Impulsiveness Scale-11, Beck Anxiety Inventory, and Beck Depression Inventory. We characterized the centrality parameters of BN, impulsiveness, and anxiety and depression symptoms of BN patients compared with HCs.

**Results:**

Among all symptoms in the constructed BN group network, Shape dissatisfaction had the highest strength. In the BN group network, three clusters of symptoms (“ED-specific symptoms,” “impulsivity,” and “anxiety and depression”) were linked to each other by several symptoms. Compared to the HC network, impulsiveness was strongly associated with Concerns about Others Seeing One Eat in the BN network.

**Conclusion:**

This study shows that ED-specific symptoms, i.e., Shape dissatisfaction, play a key role in BN. The cognition of “shape dissatisfaction” is a basis, and impulsivity and emotional symptoms are maintaining factors that may lead to BN development.

## Introduction

Bulimia nervosa (BN) (1) is a severe and complex eating disorder (ED). BN is mainly characterized by fear of weight gain, excessive preoccupation with body weight and shape, recurrent binge eating and subsequent purging behaviors, which are common in adolescents and young women (2). Clinically, 90–95% of BN patients are female (3). Lifetime estimates of BN diagnosed according to the Diagnostic and Statistical Manual of Mental Disorders (DSM-5) are between 4 and 6.7% (4).

Patients with BN often show impaired inhibitory control. Previous studies have demonstrated that individuals with BN have high trait impulsivity (5, 6). Patients with BN act on impulse and without planning. In addition, impulsive traits are related to suicide attempts and non-suicidal self-injury (NSSI) in BN patients (7). Thus, impulsive traits may be a risk factor related to BN. Patients with BN are frequently comorbid with depression and anxiety (1). Under stress, patients with BN easily generate negative affect (such as anger and sadness), and they may engage in impulsive action (i.e., binge eating, NSSI) to alleviate negative emotions (8). Therefore, emotional state may be another risk factor for BN (9). Impulsivity and depression/anxiety may have different roles in the development and maintenance of BN. Understanding how specific facets of impulsive traits and negative affect may relate ED-specific symptoms could provide insight into this complex disease.

Network analysis is a means for understanding the complex interactions that occur in diseases. It has been applied to clinical psychology in recent years, with an advantage in that it can provide insight into the roles of various symptoms in mental disorders (10). An important goal of the network analysis technique is to identify the central symptoms in the network, which are thought to be highly influential symptoms. Because high-centrality symptoms can be activated by other symptoms in the network, they have strong associations with other symptoms (11). In general, identifying high-centrality symptoms may help identify targets for clinical intervention (12–14).

In recent years, several network analyses have been carried out in patients with BN (15–18). Previous studies have focused more specifically on ED symptoms; for example, Overvaluation of Weight and Shape (15), Shape and Weight Preoccupation (16), Fear of Weight Gain (17), Desiring Weight Loss, Restraint and Worries that the feeling will get out of control, and Guilt after overeating (18) were the most important ED symptoms. Previous network analyses studies focused more attention on cognitive bias in weight and shape. Although a previous network study with a large sample of BN patients focused on emotional states and personal traits, it did not focus on impulsivity (19). Nevertheless, impulsivity and anxiety/depression also play key roles in BN development and progression. However, little is known about how ED-specific symptoms, impulsive traits and mood states interact with each other in this complex system. It is necessary to focus on personality traits, emotional states and the relationship between impulsive traits/emotional states and the ED-specific symptoms of BN. This will be helpful to BN patients in developing new treatments and promoting recovery.

The main aim of the present study was to utilize a network analysis approach to identify the central symptoms of BN. We compared networks of individuals with BN and healthy controls (HCs) and analyzed the differences between the two networks to understand the relationships between ED symptoms, impulsivity, anxiety and depression. We hope to provide a theoretical basis for novel therapeutic interventions by identifying the relationship between ED symptoms and the abovementioned risk and perpetuating factors. The hypotheses of the study were as follows: (a) ED-specific symptoms, i.e., Shape/weight dissatisfaction, Fear of Weight Gain and Desire to lose weight, are more central to the BN network; and (b) the ED-specific symptoms, impulsivity and emotional symptoms influence one another, which contribute to the development and maintenance of this disease.

## Materials and Methods

### Participants

This study was a retrospective and cross-sectional observational study. We included BN patients who were recruited from the outpatient and clinical psychological wards of Shanghai Mental Health Center from January 2019 to December 2020. All patients were diagnosed by certified psychiatrists using the Diagnostic and Statistical Manual of Mental Disorders, 5th edition (DSM-5). Patient information (including age, BMI, years of education, etc.) is provided in Table 1. The exclusion criteria were as follows: (1) subjects with other psychiatric comorbidities (e.g., substance dependence in the last 3 months, schizophrenia, bipolar I disorder, current significant suicidal ideation or behaviors); (2) subjects with cognitive impairment or a history of brain trauma or brain disease; and (3) the researchers considered participation in this study inappropriate for other reasons (e.g., patients who could not understand the content of the questionnaire, patients unable to comply with the requirements of the tests). The HCs were recruited by advertising. The HC subjects did not currently or previously meet any psychiatric diagnosis criterion. The HC participants were matched to the BN patients according to various characteristics. This study complies with ethical standards, and it was approved by the Ethics Committee of Shanghai Metal Health Center (Ethical Approval No. 2020–14). Informed consent was obtained from all participants before any study procedure was conducted.

**TABLE 1:** Sample demographics and symptom descriptions.

	BN (*n* = 146) (Mean ± SD)	HC (*n* = 146) (Mean ± SD)	*t*-value	*p*-value
Age	20.84 ± 2.70	20.93 ± 2.62	−0.29	***0.775***
Education year	14.25 ± 2.39	14.08 ± 2.86	0.58	***0.564***
BMI	20.78 ± 5.44	21.45 ± 6.19	−0.99	***0.323***
**EDE-Q^†^**				
Restriction	3.11 ± 1.89	0.69 ± 0.93	13.87	**<*0.001***
Eating concern	3.43 ± 1.37	0.39 ± 0.63	24.37	**<*0.001***
Shape concern	4.33 ± 1.48	1.47 ± 1.31	17.51	**<*0.001***
Weight concern	3.95 ± 1.51	1.29 ± 1.25	16.37	**<*0.001***
Total score	3.71 ± 1.33	0.96 ± 0.91	20.63	**<*0.001***
**BIS-11^†^**				
Attention impulsiveness	14.49 ± 3.63	5.80 ± 6.45	14.19	**<*0.001***
Motor impulsiveness	19.51 ± 5.03	8.44 ± 9.38	12.57	**<*0.001***
Unplanned impulsiveness	24.56 ± 5.99	10.71 ± 12.05	12.44	**<*0.001***
Total score	58.56 ± 12.17	24.95 ± 27.60	13.46	**<*0.001***
**BDI^†^**				
Total score	27.45 ± 12.09	2.64 ± 4.51	23.23	**<*0.001***
**BAI^†^**				
Total score	15.43 ± 10.95	1.16 ± 2.68	15.30	**<*0.001***

*^†^EDE-Q, The Eating Disorders Examination Questionnaire, Chinese version; BIS-11, The Barratt Impulsiveness Scale (version 11, BIS-11); BDI, Beck Depression Inventory; The bold values are BAI, Beck Anxiety Inventory. n, number of samples; t, t-value; p, p-value.*

### Measures

#### Eating Disorder Examination Questionnaire 6.0

The EDE-Q 6.0 is a widely used 28-item self-report questionnaire that assesses the main behavioral and psychological characteristics of eating disorders over the previous 4 weeks (20, 21) using a 7-point Likert-type scale (0: never; 6: every day). The Chinese version had excellent internal consistency (Cronbach’s α = 0.95) (22). The EDE-Q includes four subscale scores, namely, Restriction, Eating concern, Shape concern and Weight concern. The EDE-Q generates two types of data. First, 22 items (items 1–12 and 19–28) reflect the severity of ED symptoms. Second, 6 items (items 13–18) provide data on the six main behavioral characteristics of ED in terms of presence/absence and frequency with which the behavior occurred and loss of control. The scores of the subscales are obtained by calculating the average of the items of each subscale, and the global score (EDE-Q 6.0) is the average of scores of the four subscales.

#### Barratt Impulsiveness Scale (Version 11)

The BIS-11 is one of the most widely used self-report measurements of impulsivity (23). It consists of 30 items measured on a 4-point Likert scale (1 = rarely/never, 2 = occasionally, 3 = often, 4 = almost always/always), with 11 items reverse scored. The BIS-11 is divided into three dimensions: attentional impulsiveness (AI), motor impulsiveness (MI), and nonplanning impulsiveness (NPI). The scale has been translated into Chinese, and the internal consistency of the 30-item scale and the three 10-item subscales are excellent (Cronbach’s α = 0.77-0.89) (24). The total score for the BIS-11 ranges from 30 to 120. The higher the score is, the higher the impulsiveness.

#### Beck Depression Inventory II

Depression symptoms were assessed with the BDI-II (25, 26), a 21-item self-report measure of depression. Each item is scored on a 4-point Likert scale, indicating the intensity level of symptoms. The higher the score is, the higher the depression. Studies have shown that the Chinese version of the BDI-II scale has good internal consistency (Cronbach’s α = 0.94) (27).

#### Beck Anxiety Inventory

The BAI is a 21-item self-report scale for assessing symptoms of anxiety (28, 29). Each symptom is scored on a 4-point scale rating severity from “not at all” to “severely, it bothered me a lot.” The Chinese version of the BAI has been verified to have excellent internal consistency (Cronbach’s α = 0.95) (30).

### Statistical Analyses

To ensure data quality, records were screened for inappropriate responses and a lack of response variation to open-ended questions. The descriptive analysis of demographic information and scale measures was performed with SPSS version 26.0 software. The scores of the above scales were compared by using two-tailed independent *t-*tests between the BN and HC groups, with the significance level set as 0.05.

### Network Estimation

Network models were constructed and analyzed with R software (version 4.05) using the qgraph package (31, 32).

The partial correlation network method was used to estimate all symptom networks, and the edges of the network can be understood as correlations between symptoms after adjusting all other edges. The procedure for estimating each partial correlation network was as follows. First, we used a Gaussian graph model to estimate the pairwise correlation parameters between nodes (33). As parameter estimation of all edges can lead to type I error, we controlled this by using a least absolute shrinkage and selection operator (LASSO) (34) to create a more parsimonious network by reducing small correlations to exactly zero (34, 35). This step was embedded in a best model selection procedure, in which the extended Bayesian information criteria (EBIC) were minimized.

In this network, a circle represents an individual symptom (one item from the symptom measures) from the EDE-Q 6.0, three subscales of BIS-11 or BDI and the BAI total score. The associations between nodes are represented with lines (or “edges”). “Edges” are lines between nodes representing regularized partial correlations. The existence of edges represents dependencies between variables; blue edges indicate positive associations, and red edges indicate negative associations. The wider the edge is, the stronger the association. Abbreviations were used to designate each of the 28 EDE-Q 6.0 items and the three subscales of the BIS-11 in the study (see Supplementary Table 1). These abbreviations are used in figures depicting the centrality values of nodes.

### Network Centrality Estimation

We employed the three common measures of centrality measures, i.e., “strength,” “closeness,” and “betweenness,” to quantify the features of the nodes (36). “Strength” represents the total weights of connections from other nodes to a specific node. “Closeness” is defined as the inverse of the sum of the shortest distances from a particular node to all other nodes in the network, whereby the shortest distance is the minimal number of edges traversed from one node to the next. High closeness indicates that the average distance between a given node and all other nodes in the network is short. “Betweenness” is the number of times that the shortest path between any two symptoms passes through another. A node high in “betweenness” can be regarded as a “bridge” that connects other symptoms; that is, if a high node is removed, the distances among other nodes will generally increase (37).

### Network Accuracy and Stability Estimation

The accuracy of edges and stability estimates for the network were calculated using a bootstrapping procedure with 1000 iterations (32). First, we estimated the accuracy of the edge through the 95% confidence interval (CI) of the bootstrap edge weight, with a narrower edge weight CI denoting higher accuracy. Second, we tested the stability of the centrality by subset bootstrapping. We estimated the centrality stability coefficient (CS-coefficient) as a reference index. For the CS-coefficient, values below 0.25 indicate unstable strength, and values greater than or equal to 0.5 are recommended (32).

### Network Comparison

To explore the possible difference in global connectivity and to examine the differences in network structure between the BN and HC groups, we compared the partial correlation network for the BN and HC samples using the NetworkComparisonTest package in R (38). Comparing networks for unequally sized groups is problematic because network analysis methods penalize/shrink edges based on sample size. A workaround for this issue is to balance group sizes using a bootstrapping/subsampling method.

## Results

### Clinical Characteristics

In this study, 250 questionnaires were distributed, and 209 were completed, with a response rate of 83.6% in the BN group; 484 questionnaires were distributed, and 479 questionnaires were effectively returned from the HC group, for a response rate of 99.0%. All questionnaires were administered online. Propensity score matching was used to match BN patients and HCs based on age. Ultimately, 146 BN patients and 146 HCs were selected for final analysis. The descriptive statistics for the sample are shown in Table 1. The EDE-Q total and four subscale scores, BIS total scores and three subscale scores, BDI total scores, and BAI total scores were significantly higher in the BN group than in the HC group. However, there was no significant difference in age, education years, or BMI between the BN group and HC group.

### Characteristics of the Symptom Networks

**FIGURE 1:**
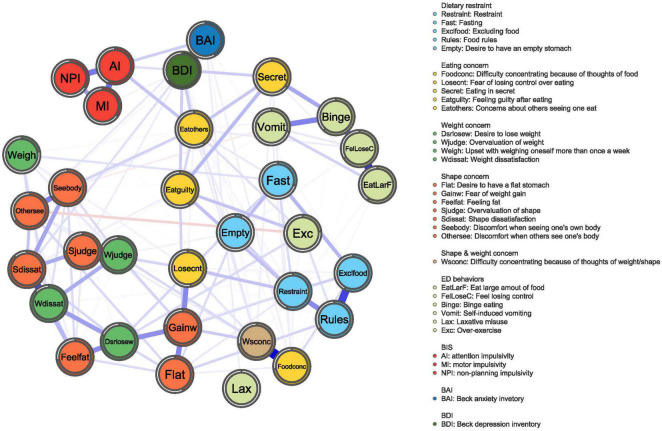
BN network. The network graph shows associations and predictability estimates between ED symptoms (i.e., fear of weight gain, Shape dissatisfaction, Weight dissatisfaction, and Restraint), depression symptoms, anxiety symptoms, and impulsivity (i.e., attention impulsiveness, motor impulsiveness, and unplanning impulsiveness) in BN. The edges represent the strength of association between nodes. The colors of the node indicate the different subscales.

**FIGURE 2:**
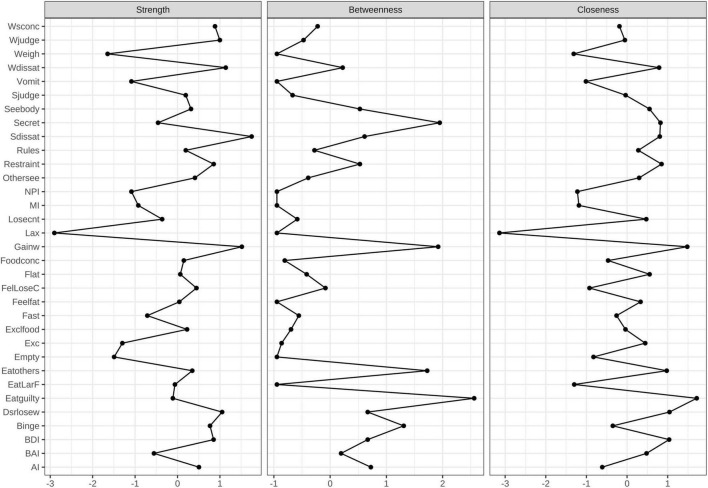
Centrality measures for the BN network representing the strength, closeness, and betweenness of each node. Higher numbers indicate that the item is more central to the network.

**FIGURE 3:**
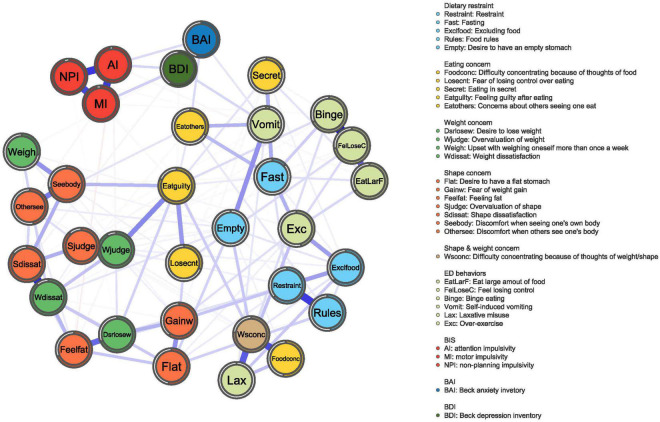
HC network. The network graph shows associations and predictability estimates between ED symptoms (i.e., feel losing control, Shape dissatisfaction, Weight dissatisfaction, Difficulty concentrating because of thoughts of weight/shape), depression symptoms, anxiety symptoms, and impulsivity (i.e., attention impulsiveness, motor impulsiveness, and unplanning impulsiveness) in HC. The edges represent the strength of association between nodes. The different colors of the node indicate the different subscales.

**FIGURE 4:**
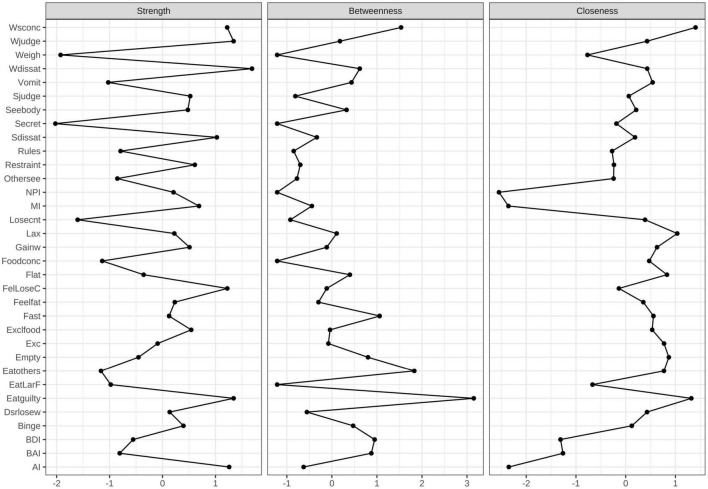
Centrality measures for the HC network representing the strength, closeness, and betweenness of each node. Higher numbers indicate that the item is more central to the network.

The network structure of the EDE-Q, three subscales of BIS-11, BDI, and BAI total scores in different groups, as well as a comparative plot centrality index of the two groups are depicted in Figures 1–4, respectively.

#### The Core Symptoms of Bulimia Nervosa Psychopathology

Figure 1 shows the constructed symptom network of BN. As illustrated in Figure 2, the highest strengths included (top 3): Shape dissatisfaction (strength = 1.31), Fear of weight gain (strength = 1.25), and Weight dissatisfaction (strength = 1.11). It is worth noting that several nodes have the highest value for “strength” measures, suggesting that these symptoms are of particular importance in the network (i.e., Shape dissatisfaction, Fear of weight gain, and Weight dissatisfaction). Shape dissatisfaction had significantly higher strength values than all other symptoms. Regarding node predictability, the mean node predictability was 53.2%; thus, on average, 53.2% of the variance in each node was explained by neighboring nodes.

Three clusters of symptoms (“ED-specific symptoms,” “impulsivity,” and “anxiety and depression”) are linked to each other by several symptoms in Figure 1. As shown, the ED-specific symptom that is closest to “impulsivity” is “Concerns about others seeing one eat.” Those closest to “anxiety and depression” symptoms are as follows: Feeling guilty after eating, Concerns about others seeing one eat, and Eating in secret.

#### Core Symptoms of Healthy Controls

Figure 3 displays the constructed network for the HCs. As indicated in Figure 4, the highest strengths include (top 3): Weight dissatisfaction (strength = 1.27), Feeling guilty after eating (strength = 1.19), and Overvaluation of weight (strength = 1.19). Weight dissatisfaction had significantly higher strength values than all other symptoms. The mean predictability of the nodes was 58.6%, which was similar to the mean node predictability of the BN group.

Eating disorder-specific symptoms had a weak correlation with both impulsivity and mood states (anxiety and depression) in the network analysis of HCs.

#### Accuracy and Stability Analysis

**FIGURE 5:**
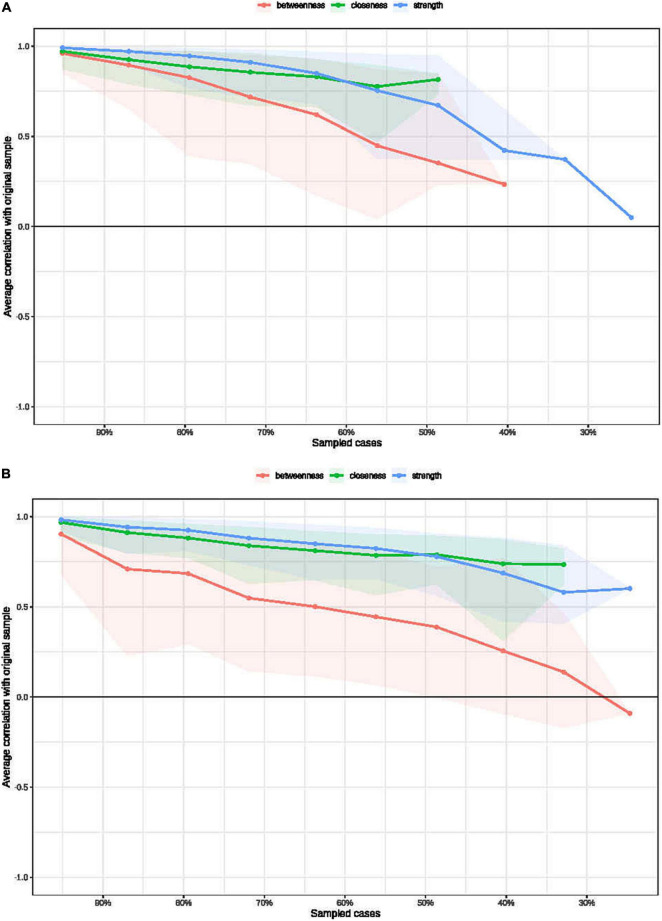
Bootstrapped stability for the BN **(A)** and HC **(B)** graphical least absolute shrinkage and selection operator networks. The *x*-axis indicates the included portion of cases, and the *y*-axis indicates the average correlations with the original samples.

Figure 5 shows the nodes used to determine centrality estimate stability (edge weight bootstrapping accuracy in the BN and HC networks, see Supplementary Figures 1, 2, respectively). The CS-coefficient indexes of the BN and HC groups were 0.28 and 0.36, respectively, and the results from the stability analyses showed that the network models were relatively stable.

#### Network Comparison Analysis

**FIGURE 6:**
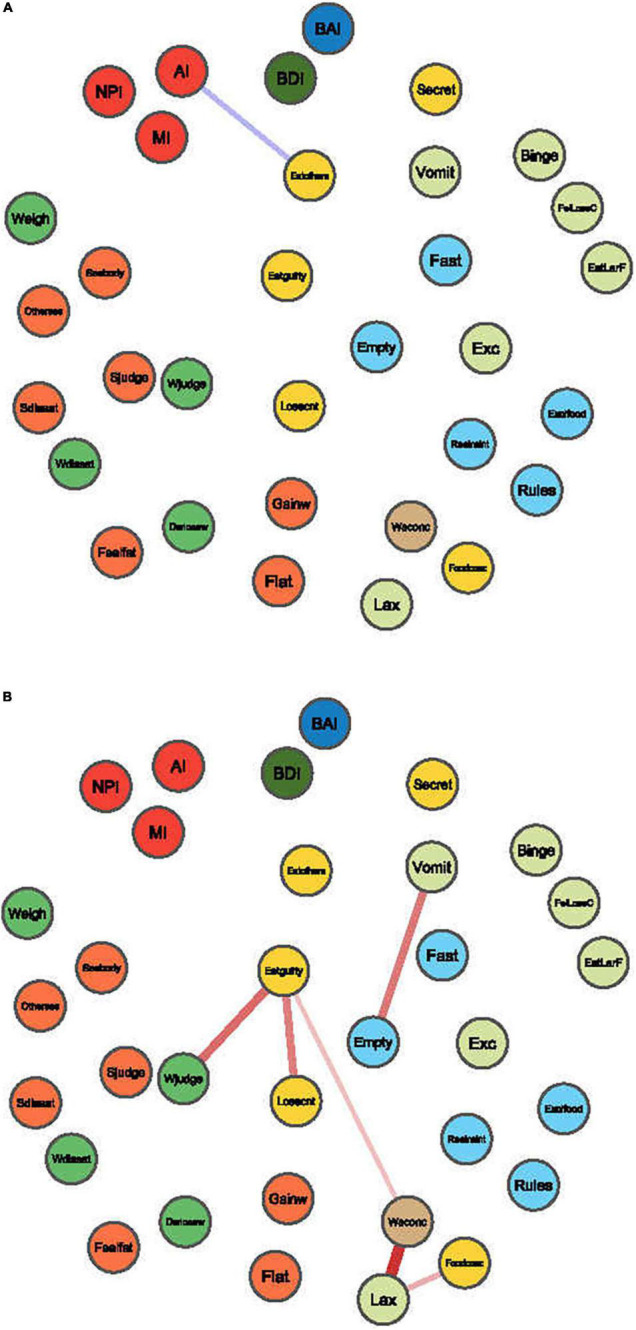
Edges exhibiting significant differences between BN and HC. The blue edges denote the increased correlations between items in the BN compared with those in the HC network **(A)**, and red edges denote the decreased correlations **(B)**.

We used the NetworkComparisonTest package to compare differences in edge weights between the BN and HC networks, and the edge weight was false discovery rate (FDR)-adjusted. Significant positive and negative correlations are shown in Figure 6. The global strength difference between the BN network (global strength = 13.75) and HC network (global strength = 14.49) was significant (*p* = 0.01).

Compared to HCs, attentional impulsiveness on the BIS-11 was more strongly connected with Concerns about others seeing one eat (mean difference = 0.13, *p* = 0.000) on the EDE-Q (Figure 6A). In contrast, the symptom of Feeling guilty after eating showed weak connections with Overvaluation of weight (mean difference = −0.27, *p* = 0.000) and Fear of losing control over eating (mean difference = −0.24, *p* = 0.04) on the EDE-Q. Similarly, Self-induced vomiting had a weak connection with Desire to have an empty stomach (mean difference = −0.25, *p* = 0.000). Laxative misuse weakened the connectivity of Difficulty concentrating because of thoughts of weight/shape and Difficulty concentrating because of thoughts of food (mean difference = −0.38, −0.15, and −0.12; *p* = 0.000, 0.000, respectively) on the EDE-Q (Figure 6B).

## Discussion

This study identified the core ED symptoms and the relationships among ED symptoms, impulsive traits, anxiety and depression in BN patients in mainland China. First, Shape Dissatisfaction, Fear of Weight Gain and Weight Dissatisfaction had the highest strength in the BN symptom network. Second, three clusters of symptoms (“ED-specific symptoms,” “impulsivity,” and “anxiety and depression”) were linked to each other by several symptoms. Overall, generating a complex network based on the relationship between symptoms may help better understand the disease.

First, our results showed that Shape dissatisfaction, Fear of Weight Gain and Weight dissatisfaction had the highest strength in the BN network, with Shape dissatisfaction having the highest strength value. Shape dissatisfaction and Fear of Weight Gain are part of the “Shape concern” subscale, and Weight dissatisfaction is part of the “Weight concern” subscale. Shape concern and Weight concern involve dissatisfaction with one’s own body shape and weight, desire to control weight, and preoccupation with shape and weight. These symptoms are core ED-specific symptoms in patients with BN. Previous cross-sectional studies also found similar results in patients with BN (15–17, 19, 39). These findings show that Shape dissatisfaction, Fear of Weight Gain and Weight dissatisfaction are central to BN psychopathology, and ED thinking rather than ED behaviors plays a central role in BN (4).

Second, the results also showed that some symptoms on the EDE-Q were connected to anxiety/depression symptoms and impulsivity in the BN group. Patients with BN are often described as being in a “vicious cycle”: BN patients are concerned about weight, shape and strict dietary restrictions. Then, they experience physical and psychological cravings for food, break dietary rules, think that they have failed and feel out of control and guilty. Finally, they compensate by binge-fasting, purging, and exercise and are more focused on weight and shape (4). Hence, in this state, they often experience anxiety and depression. Moreover, BN patients are associated with emotional regulation problems. During the COVID-19 lockdown, BN patients experienced exacerbated binge eating because of difficulties in emotional regulation (40). When they are in a bad mood, they are unable to adopt positive methods to deal with their emotions instead of using impulsive behaviors, such as episodic binge eating, to relieve their emotions, which aggravates their emotional problems (41). Solmi et al. suggested that core ED symptoms, depression and anxiety symptoms play an important role in BN (19). However, their studies did not focus on impulsivity traits. Our results were consistent with those of a previous study showing that ED-specific symptoms and emotional state occupied a major position. In addition, our study suggests that impulsivity traits may be another key factor. In short, ED-specific symptoms, i.e., Concern about weight and shape and Fear of gain weight are the foundation of BN. Impulsive traits and negative mood are maintenance factors of BN. These factors influence each other in the BN network. However, this study missed information on comorbidities. Anxiety and depression are co-occurring diseases of BN needing treatment. These issues require more attention in future research.

Third, our study indicated that the total score and three subscale scores of the BIS-11 were increased in the BN group compared with the HC group. Our study also found that in the BN network, attentional impulsiveness had a stronger connection with Concerns about others seeing one eat on the EDE-Q than that in the HC network. These findings suggest that the greater impulsiveness in BN patients is extensive and non-specific. Attentional impulsiveness includes two factors: attention and cognitive instability. Attentional impulsiveness is correlated with both proactive and reactive control (42). Dorsolateral prefrontal cortex (DLPFC) areas are associated with impulsivity (43). Interestingly, the DLPFC also plays an important role in emotion regulation (44). In recent years, dialectical behavioral therapy (DBT) has become a good treatment for emotional eating in BN (45). DBT is a behavioral treatment that draws its principles from behavioral science, dialectic philosophy, and Zen practice, including mindfulness and distress tolerance (46). Furthermore, some studies have shown that novel non-invasive neuromodulation therapy, such as transcranial magnetic stimulation (TMS), can improve impulsiveness (43). It is directed at modulating activity in brain regions (i.e., the DLPFC) and may also be used as an alternative intervention for BN in the future.

Several limitations should also be considered. First, the current study involved cross-sectional data without longitudinal follow-up; a cross-sectional study cannot distinguish symptoms that change over time or infer the directionality of relationships between ED-specific symptoms, impulsiveness and emotional symptoms. Future research should focus on properly designed prospective cohort studies, and longitudinal designs will elucidate treatment-based symptom changes in BN networks. Second, a previous study showed that patients with binge eating disorder (BED) have regular binge eating behaviors. BED is regarded as multifactorial, with a special focus on several neurocognitive deficits in executive functioning, such as inhibitory control and attentional bias. The present study did not explore the effect of these intriguing variables, which requires further investigation in future studies. Finally, information on relevant comorbidities was missing. These covariates may have influenced the analyzed outcome variables.

## Conclusion

This network analysis assessed ED-specific symptoms, impulsive personality traits, anxiety and depression in a sample of patients with BN in mainland China. This study was matched by age, which reduces possible confounding by these factors. In addition, we included some potentially important risk factors, such as impulsivity and emotion-related variables (BDI and BAI). The study found that Shape Dissatisfaction, Fear of Weight Gain, and Weight Dissatisfaction are the core symptoms in the BN network. There was a relationship between impulsiveness, anxiety/depression symptoms and ED-specific symptoms. Thus, the results will provide a potential theoretical basis for new intervention measures, such as DBT and TMS, in the future.

## Data Availability Statement

The raw data supporting the conclusions of this article will be made available by the authors, without undue reservation.

## Ethics Statement

The studies involving human participants were reviewed and approved by the Ethics Committee of Shanghai Metal Health Center. The patients/participants provided their written informed consent to participate in this study.

## Author Contributions

JC and HZ contributed to the study concept and design. YC assisted with data analysis and wrote the manuscript. LG conducted statistical analysis and interpretation. YC, LG, MW, LZ, and QH contributed to the acquisition of the data and clinical assessment. JC, HZ, LW, and YZ contributed to a critical revision of the manuscript for important intellectual content. All authors reviewed the manuscript and approved the final version.

## Conflict of Interest

The authors declare that the research was conducted in the absence of any commercial or financial relationships that could be construed as a potential conflict of interest.

## Publisher’s Note

All claims expressed in this article are solely those of the authors and do not necessarily represent those of their affiliated organizations, or those of the publisher, the editors and the reviewers. Any product that may be evaluated in this article, or claim that may be made by its manufacturer, is not guaranteed or endorsed by the publisher.
