# Supplementary material for: Network Analysis of Eating Disorders Symptoms Co-occurring With Impulsive Personality Traits and Negative Mood States in Patients With Bulimia Nervosa
Source: Front Psychiatry. 2022 May 18;13:899757. doi: 10.3389/fpsyt.2022.899757 (PMC9157589; doi:10.3389/fpsyt.2022.899757)
Supplement: Supplementary file 1 [file Table_1.DOCX]

supplement


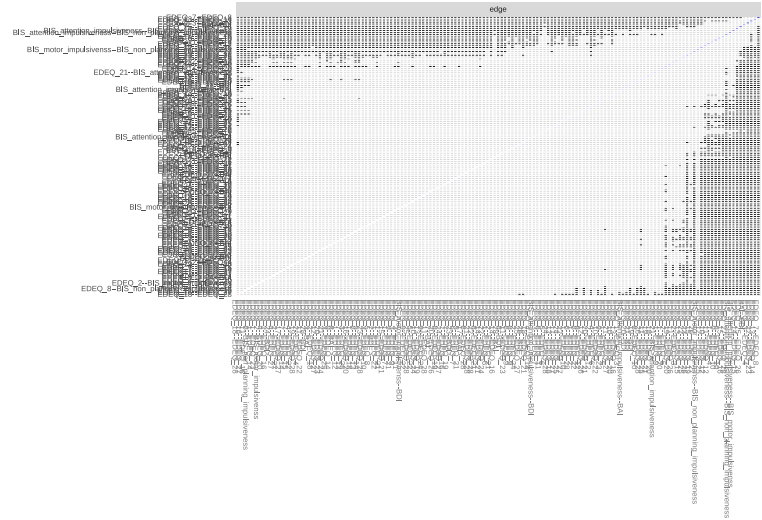


Figure 1. BN edge weight accuracy bootstrapping results. Black boxes indicate significant strength differences, meaning that the bootstrapped strength difference confidence interval does not span 0. Gray boxes indicate nonsignificant strength differences, meaning that the bootstrapped strength difference confidence interval spans 0.


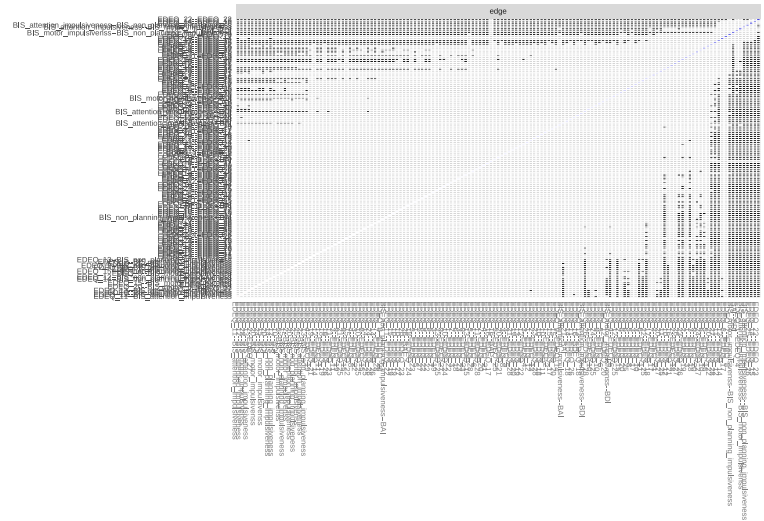


Figure 2. HC edge weight accuracy bootstrapping results. Black boxes indicate significant strength differences, meaning that the bootstrapped strength difference confidence interval does not span 0. Gray boxes indicate nonsignificant strength differences, meaning that the bootstrapped strength difference confidence interval spans 0

Table 1. Abbreviations for EDE-Q items and 3 subscales of BIS-11

| Abbreviations | Item |
| --- | --- |
| Restraint | Restraint |
| Fast | Fasting |
| Exclfood | Excluding food |
| Rules | Food rules |
| Empty | Desire to have an empty stomach |
| Flat | Desire to have a flat stomach |
| Foodconc | Difficulty concentrating because of thoughts of food |
| Wsconc | Difficulty concentrating because of thoughts of weight/shape |
| Losecnt | Fear of losing control over eating |
| Gainw | Fear of weight gain |
| Feelfat | Feeling fat |
| Dsrlosew | Desire to lose weight |
| EatLarF | Eat large amount of food |
| FelLoseC | Feel losing control |
| Binge | Binge eating |
| Vomit | Self-induced vomiting |
| Lax | Laxative misuse |
| Exc | Over-exercise |
| Secret | Eating in secret |
| Eatguilty | Feeling guilty after eating |
| Eatothers | Concerns about others seeing one eat |
| Wjudge | Overvaluation of weight |
| Sjudge | Overvaluation of shape |
| Weigh | Upset with weighing oneself more than once a week |
| Wdissat | Weight dissatisfaction |
| Sdissat | Shape dissatisfaction |
| Seebody | Discomfort when seeing one’s own body |
| Othersee | Discomfort when others see one’s body |
| AI | BIS_attention_impulsiveness |
| MI | BIS_motor_impulsiveness |
| NPI | BIS_non_planning_impulsivensee |
