# Supplementary material for: Network Analysis of Eating Disorders Symptoms Co-occurring With Impulsive Personality Traits and Negative Mood States in Patients With Bulimia Nervosa
Source: Front Psychiatry. 2022 May 18;13:899757. doi: 10.3389/fpsyt.2022.899757 (PMC9157589; doi:10.3389/fpsyt.2022.899757)
Supplement: Supplementary file 2 [file Table_2.DOCX]

supplement

The results of original data described as follows:

Table 1. Adjusted Means and Standard Error of Mean for Sample demographics and symptom descriptive as a Covariate

|  | BN(n=209)  (Mean ± SD) | HC(n=479)  (Mean± SD) | *t* value | *p* value |
| --- | --- | --- | --- | --- |
| age | 22.57±4.25 | 19.36±2.10 | 13.15 | ***<0.001*** |
| education year | 14.79±2.69 | 12.64±2.60 | 9.64 | ***<0.001*** |
| BMI | 20.88±4.93 | 21.05±5.09 | -0.38 | ***0.704*** |
| **EDE-Q** |  |  |  |  |
| restriction | 2.93±1.83 | 0.88±1.11 | 18.00 | ***<0.001*** |
| eating concern | 3.30±1.36 | 0.56±0.76 | 33.71 | ***<0.001*** |
| shape concern | 4.18±1.51 | 1.80±1.40 | 20.07 | ***<0.001*** |
| weight concern | 3.84±1.55 | 1.61±1.36 | 18.94 | ***<0.001*** |
| total score | 3.56±1.33 | 1.21±1.01 | 25.41 | ***<0.001*** |
| **BIS-11** |  |  |  |  |
| attention impulsiveness | 14.29±3.40 | 3.54±5.77 | 25.09 | ***<0.001*** |
| motor impulsiveness | 19.52±4.57 | 5.09±8.28 | 23.66 | ***<0.001*** |
| unplanned impulsiveness | 24.50±5.67 | 6.59±10.77 | 22.71 | ***<0.001*** |
| total score | 58.31±11.23 | 15.23±24.60 | 24.24 | ***<0.001*** |
| **BDI** |  |  |  |  |
| total score | 25.38±12.71 | 1.81±3.91 | 36.84 | ***<0.001*** |
| **BAI** |  |  |  |  |
| total score | 14.25±10.96 | 1.11±3.90 | 23.13 | ***<0.001*** |

† EDE-Q: The Eating Disorders Examination Questionnaire, Chinese version

BIS-11: The Barratt Impulsiveness Scale (version 11, BIS-11)

BDI: Beck Depression Inventory

BAI: Beck Anxiety Inventory


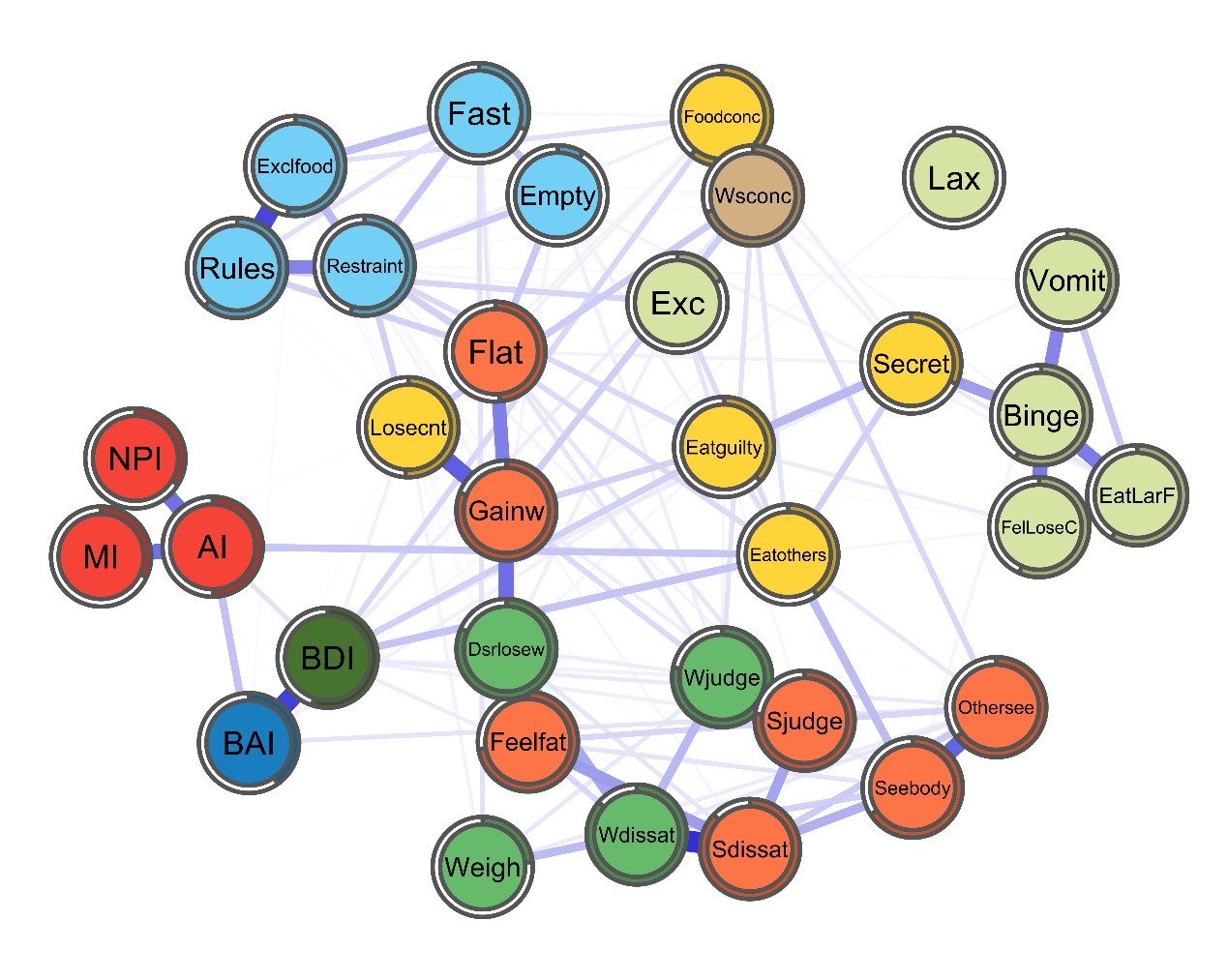


Figure 1. BN network. The network graph shows associations and predictability estimates between ED symptoms (i.e., fear of weight gain, Shape dissatisfaction, Weight dissatisfaction, Restraint), depression symptoms, anxiety symptoms, and impulsivity (i.e., attention impulsiveness, motor impulsiveness, and non-planning impulsiveness) in BN. The edges represent the strength of association between nodes. The different colors of the node indicate the different subscales.


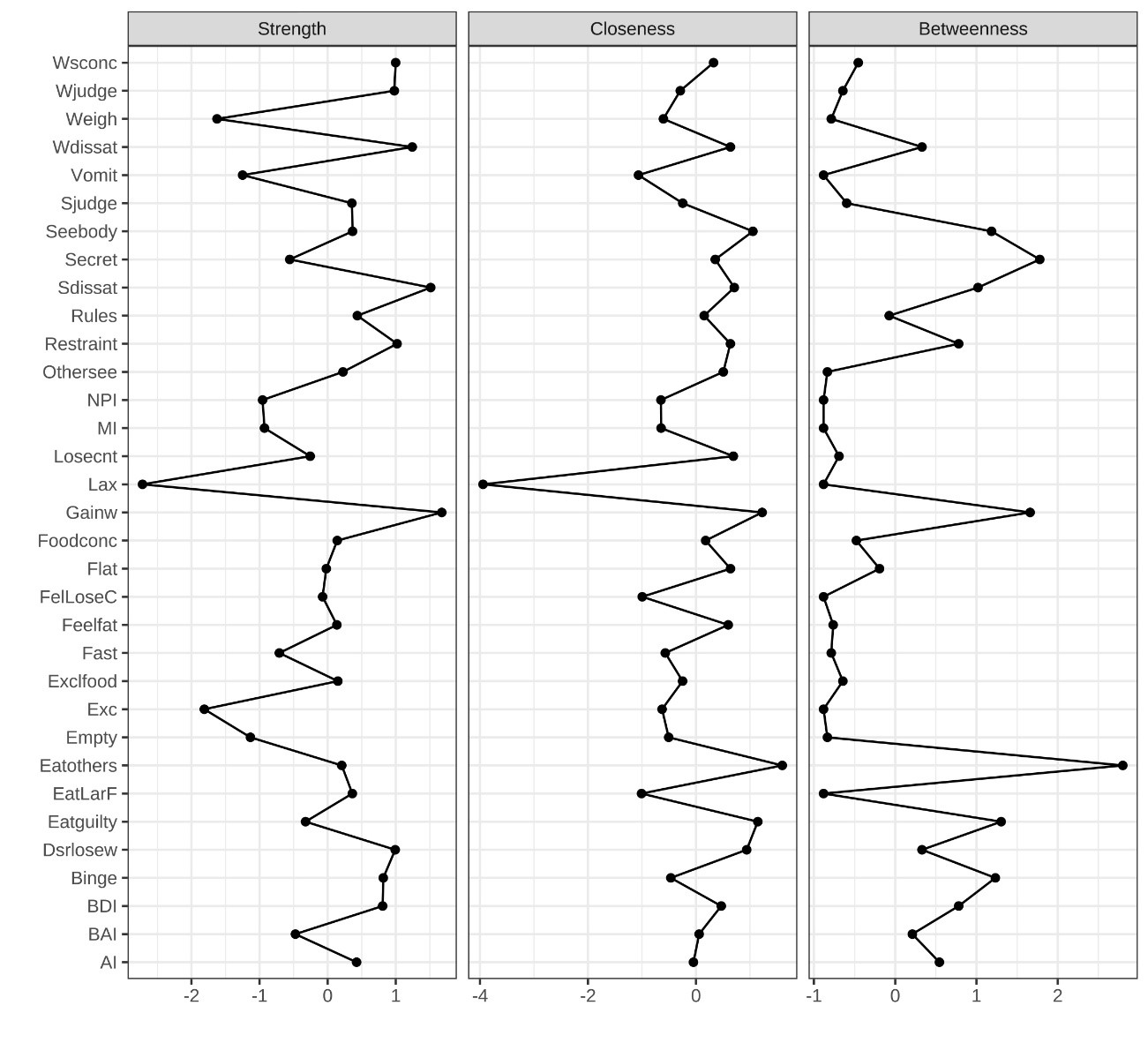


Figure 2. Centrality measures for the BN network representing the strength, closeness, and betweenness of each node. Higher numbers indicate that the item is more central to the network.


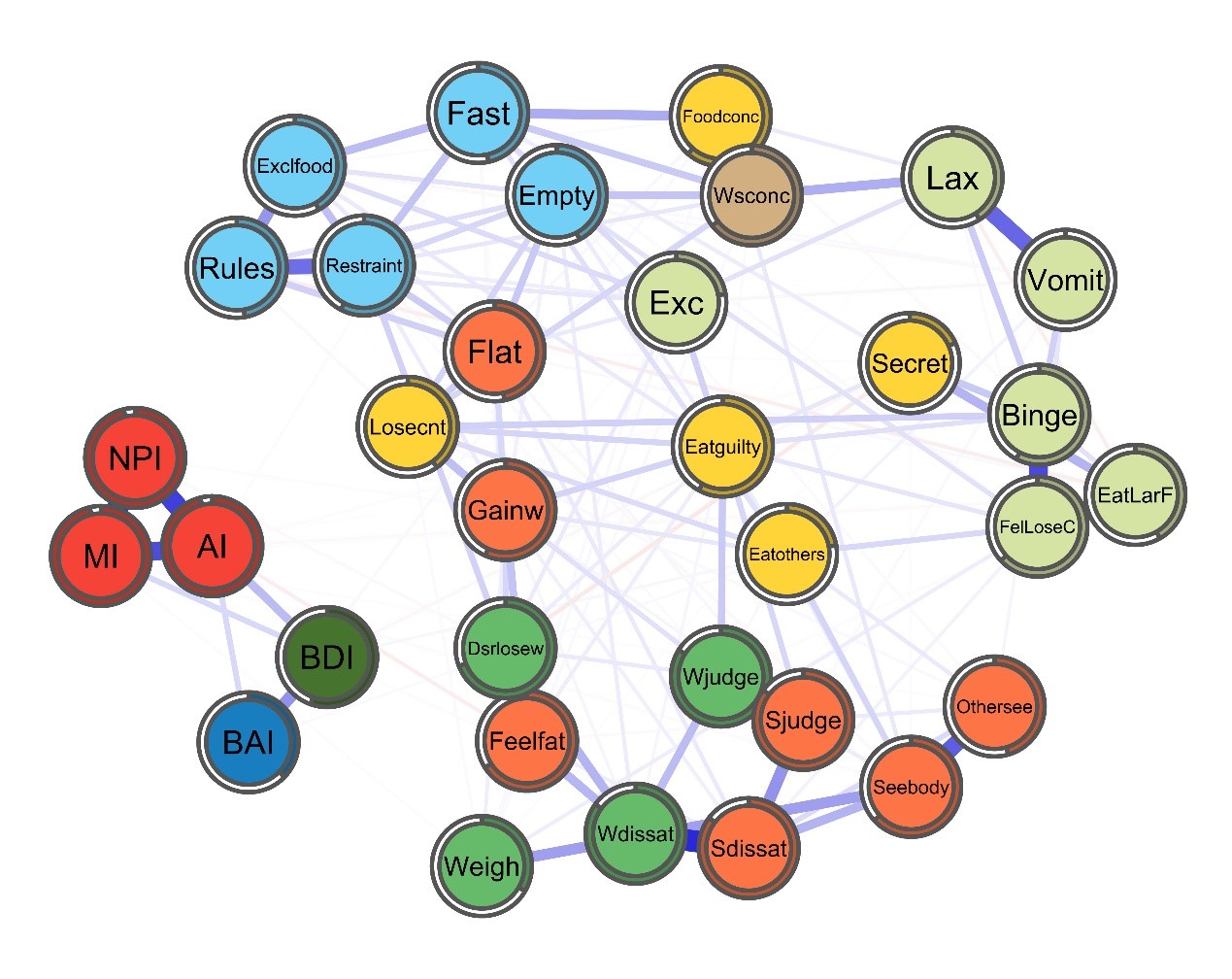


Figure 3. HC network. The network graph shows associations and predictability estimates between ED symptoms (i.e., feel losing control, Shape dissatisfaction, Weight dissatisfaction, Difficulty concentrating because of thoughts of weight/shape), depression symptoms, anxiety symptoms, and impulsivity (i.e., attention impulsiveness, motor impulsiveness, and non-planning impulsiveness) in HC. The edges represent the strength of association between nodes. The different colors of the node indicate the different subscales.


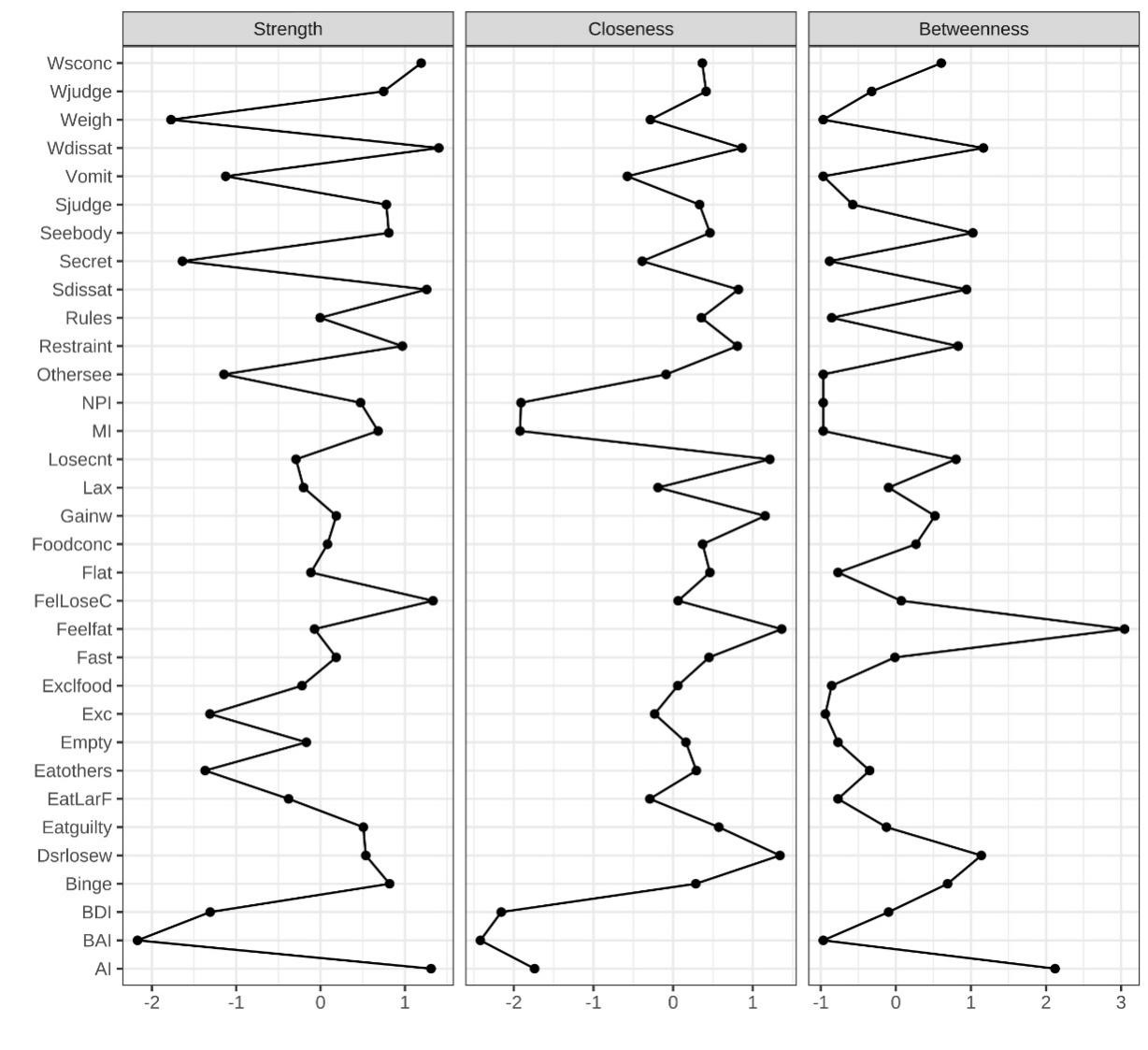


Figure 4. Centrality measures for the HC network representing the strength, closeness and betweenness of each node. Higher numbers indicate that the item is more central to the network.


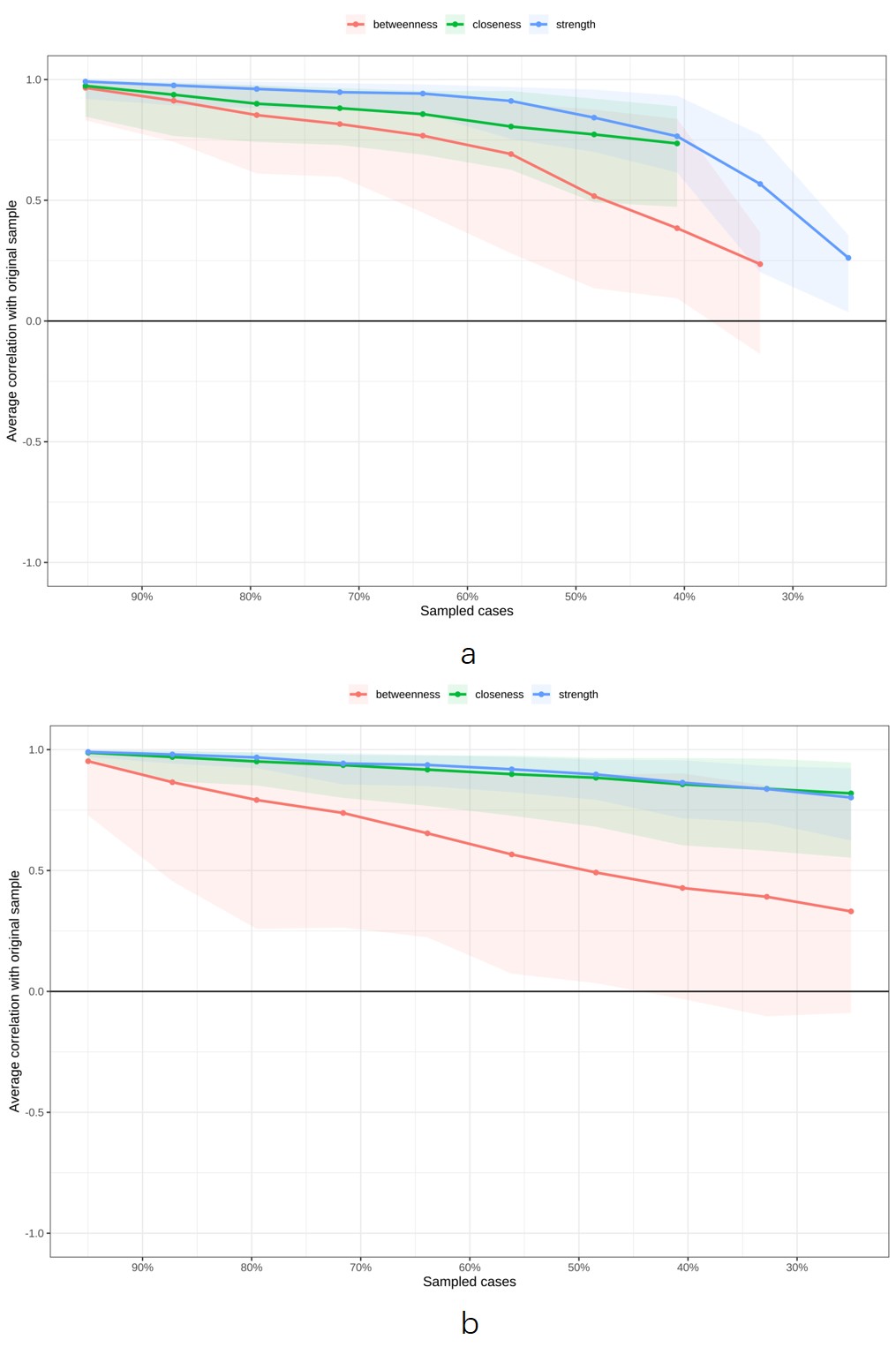


Figure 5. Bootstrapped stability for the BN(a) and HC(b) graphical least absolute shrinkage and selection operator networks. The x-axle indicates the included portion of cases, the y-axle indicates the average correlations with the original samples.


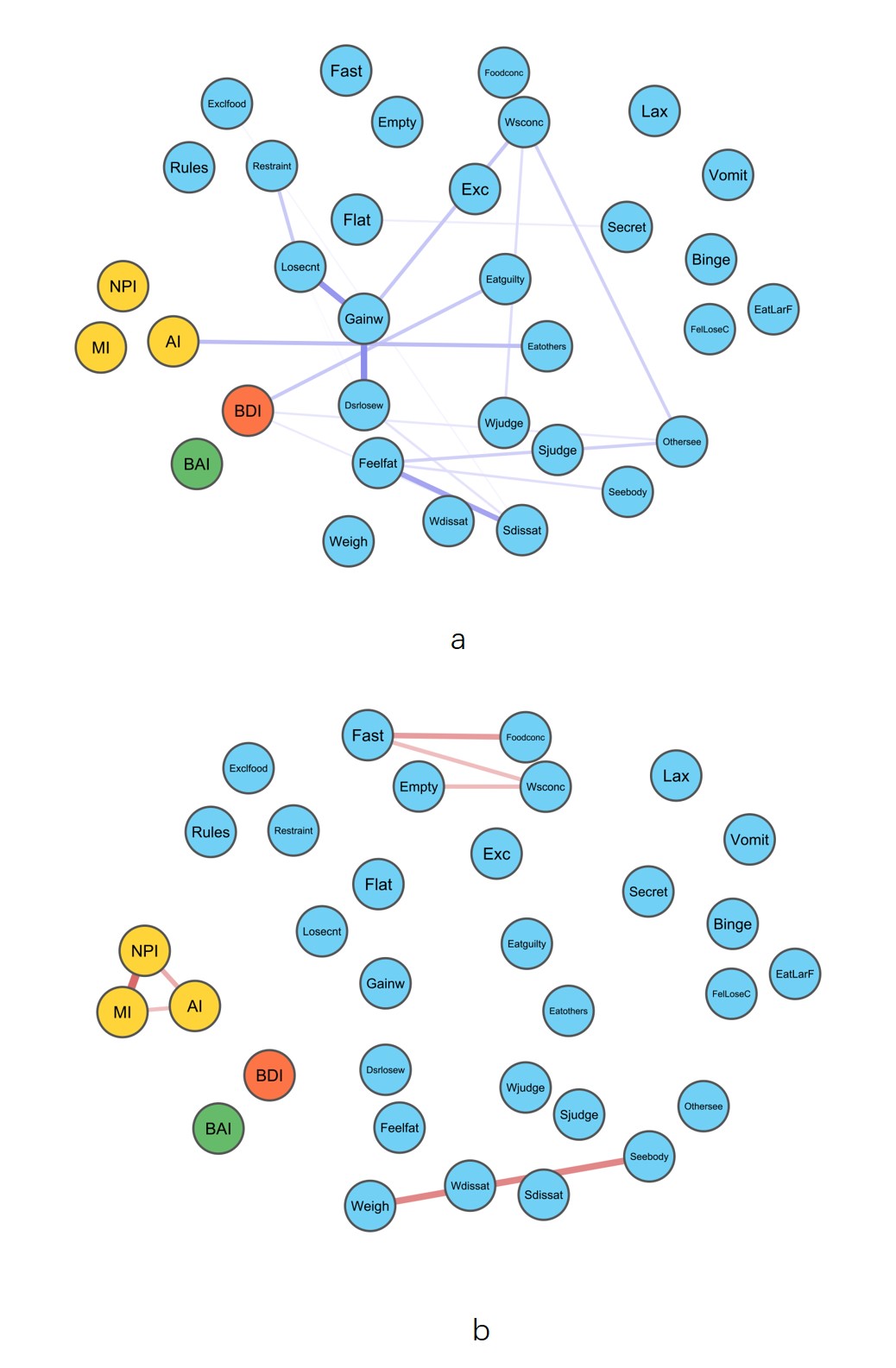


Figure 6. Edges exhibiting significant differences between BN and HC. The blue edges denote the increased correlations between items in BN compared with those in HC network(a) and red edges denote de decreased correlations(b).


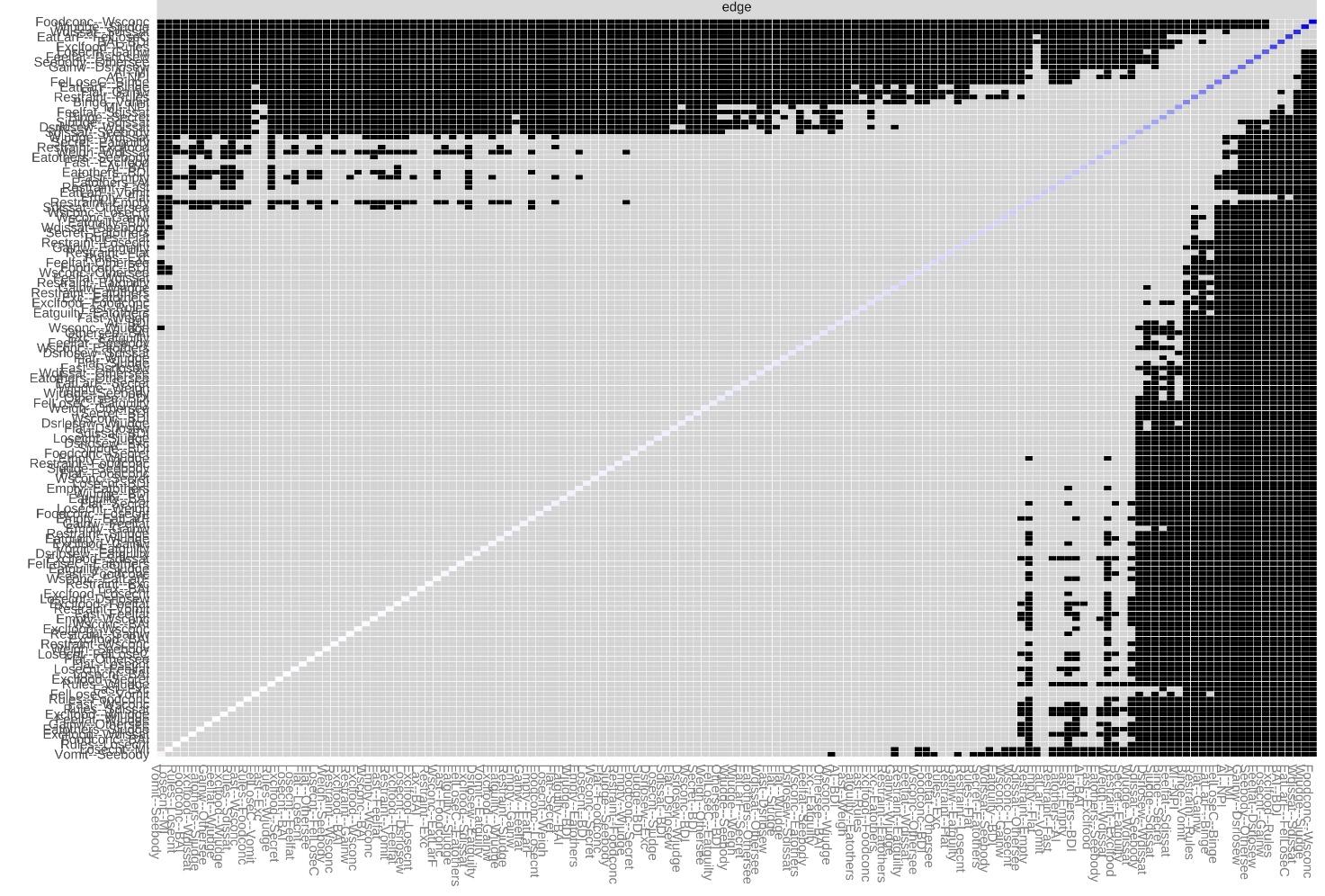


Figure 7. BN edge weight accuracy bootstrapping results. Black boxes indicate significant strength differences, meaning that the bootstrapped strength difference confidence interval does not span 0. Gray boxes indicate nonsignificant strength differences, meaning that the bootstrapped strength difference confidence interval spans 0.


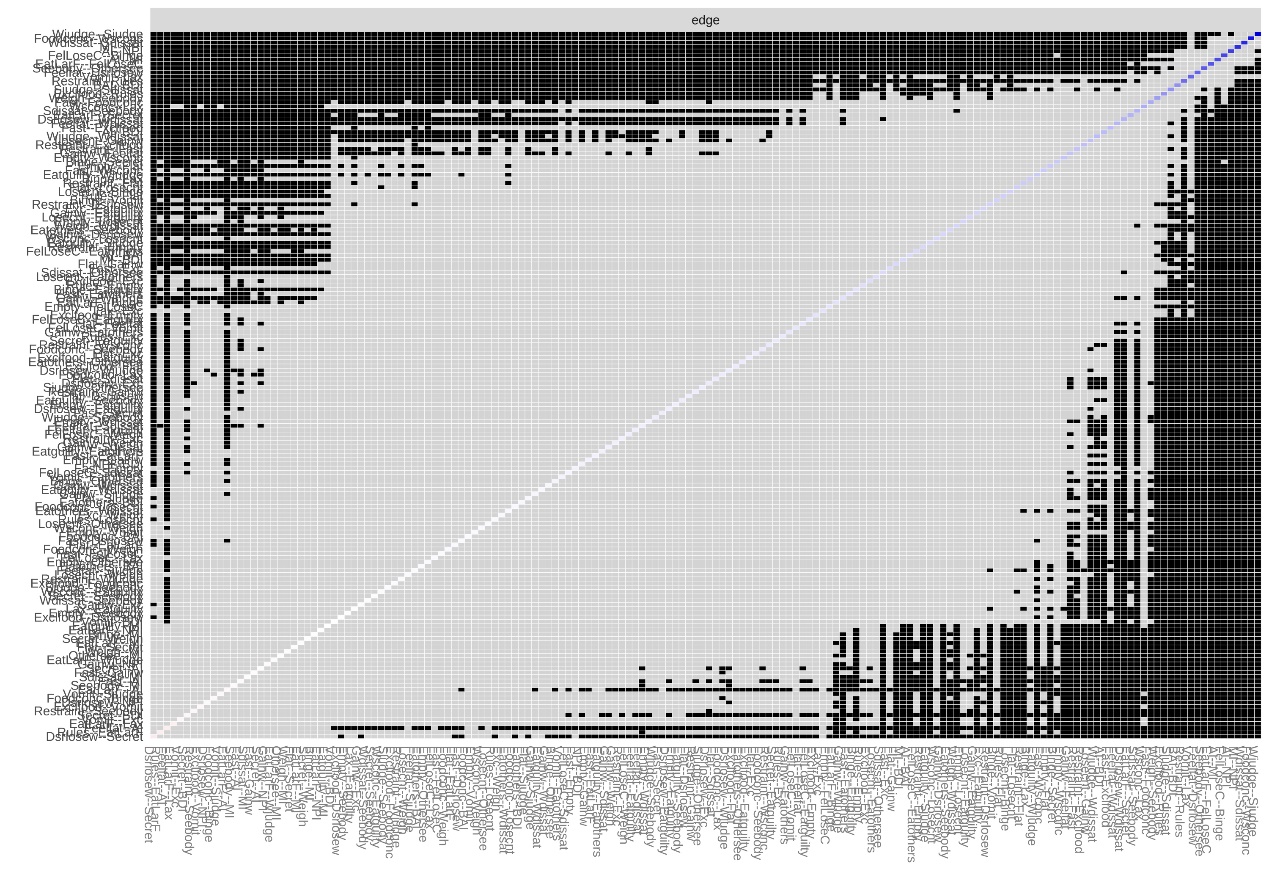
Figure 8. HC edge weight accuracy bootstrapping results. Black boxes indicate significant strength differences, meaning that the bootstrapped strength difference confidence interval does not span 0. Gray boxes indicate nonsignificant strength differences, meaning that the bootstrapped strength difference confidence interval spans 0.
